# Bacteriome-Localized Intracellular Symbionts in Pollen-Feeding Beetles of the Genus *Dasytes* (Coleoptera, Dasytidae)

**DOI:** 10.3389/fmicb.2016.01486

**Published:** 2016-09-22

**Authors:** Benjamin Weiss, Martin Kaltenpoth

**Affiliations:** ^1^Insect Symbiosis Research Group, Max Planck Institute for Chemical EcologyJena, Germany; ^2^Department for Evolutionary Ecology, Institute for Zoology, Johannes Gutenberg University of MainzMainz, Germany

**Keywords:** symbiosis, mutualism, insect, Coleoptera, pollen-feeding, intracellular

## Abstract

Several insect taxa are associated with intracellular symbionts that provision limiting nutrients to their hosts. Such tightly integrated symbioses are especially common in insects feeding on nutritionally challenging diets like phloem sap or vertebrate blood, but also occur in seed-eating and omnivorous taxa. Here, we characterize an intracellular symbiosis in pollen-feeding beetles of the genus *Dasytes* (Coleoptera, Dasytidae). High-throughput tag-encoded 16S amplicon pyrosequencing of adult *D. plumbeus* and *D. virens* revealed a single gamma-proteobacterial symbiont (‘*Candidatus* Dasytiphilus stammeri’) that amounts to 52.4–98.7% of the adult beetles’ entire microbial community. Almost complete 16S rRNA sequences phylogenetically placed the symbiont into a clade comprising *Buchnera* and other insect endosymbionts, but sequence similarities to these closest relatives were surprisingly low (83.4–87.4%). Using histological examination, three-dimensional reconstructions, and fluorescence *in situ* hybridization, we localized the symbionts in three mulberry-shaped bacteriomes that are associated with the mid- to hind-gut transition in adult male and female beetles. Given the specialized pollen-feeding habits of the adults that contrasts with the larvae’s carnivorous lifestyle, the symbionts may provision limiting essential amino acids or vitamins as in other intracellular symbioses, or they might produce digestive enzymes that break up the fastidious pollen walls and thereby contribute to the host’s nutrition. In either case, the presence of gamma-proteobacterial symbionts in pollen-feeding beetles indicates that intracellular mutualists are more widely distributed across insects with diverse feeding habits than previously recognized.

## Introduction

Many insects are associated with mutualistic microbes that represent major sources of evolutionary innovation by conveying novel ecological traits to their hosts (Douglas, 2009; Feldhaar, 2011; McFall-Ngai et al., 2013). Among the most important and widespread benefits provided by bacterial symbionts are nutritional supplementation (Douglas, 2009), degradation of fastidious polymers (Douglas, 2009; Brune, 2014), and defense against antagonists (Flórez et al., 2015). Especially in herbivorous insects, the former two can play important roles for choice and utilization of food plants (Tsuchida et al., 2004; Hosokawa et al., 2007), by allowing the insect host to specialize on nutritional resources that would otherwise be inaccessible, e.g., phloem or xylem sap or wood (Moran, 2007). As such, the acquisition of a symbiotic microbiota can enable the shift to a novel ecological niche (Sudakaran et al., 2015) and allow for subsequent adaptive radiation (Joy, 2013).

A particular feature of many obligate insect-associated symbionts is the intracellular localization within specialized organs, the so-called bacteriomes. Such structures occur across at least six different insect orders (Hemiptera, Dictyoptera, Coleoptera, Diptera, Hymenoptera, and Phthiraptera; Douglas, 1989), mostly in taxa with nutritionally challenging diets (Buchner, 1965). Concordantly, although a defensive function has recently been described for a bacteriome-localized mutualist (Nakabachi et al., 2013), such symbionts usually supplement limiting nutrients to the host (essential amino acids, B-vitamins). By contrast, involvement in digestive or detoxifying processes has usually been regarded as less likely, due to the intracellular localization separated from the gut, and direct evidence for such functions is currently lacking. However, it should be noted that the intracellular symbionts of shipworms, a group of wood-eating marine bivalves, produce digestive enzymes in the host’s gills that are transported to the gut to exert their function (O’Connor et al., 2014). Thus, even though extracellular gut bacteria appear to be predisposed toward involvement in digestion and detoxification, contributions from bacteriome-associated primary mutualists are conceivable.

Here, we investigated the microbial community associated with beetles of the genus *Dasytes* (Dasytidae). Members of this genus have long been known to harbor intracellular symbionts in mulberry-shaped bacteriomes associated with the mid-gut. After Holmgren’s (1902) initial description of the structures as “oenocytes,” Stammer (1933) realized that they are in fact clusters of bacteriocytes that are densely packed with bacterial cells. However, the identity of the symbionts, their phylogenetic affiliation, and the functional importance for the host remain unknown. The symbionts are supposedly released into the gut through short ducts (Buchner, 1965). The presence of intracellular symbionts in *Dasytes* is insofar surprising, as the beetle larvae are predaceous (Buchner, 1965) and hence unlikely to suffer from a nutritionally imbalanced or inadequate diet. The adults, however, feed on pollen, the break-down of which requires a set of plant cell wall degrading enzymes that are common among microorganisms (Shelomi et al., 2016). Hence, it is conceivable that the *Dasytes* symbionts contribute to their host’s nutrition through the production of enzymes that aid in the digestion of the pollen walls and hence make the interior nutrients available, in addition to providing a carbon and energy source for symbionts and host.

In this study, we provide a molecular characterization of the microbial community associated with *Dasytes plumbeus* and *D. virens*, as a first step toward understanding the *Dasytes* symbiosis. Furthermore, we describe the morphology and ultrastructure of the symbiont-bearing organs.

## Materials and Methods

### Ethical Statement

No permits were required for the collection of and experiments with insect specimens.

### Collection of Specimens

Adult individuals of *Dasytes plumbeus* were collected on flowers in the vicinity of Nennsdorf (Jena), Germany, on July 11, 2013, and fixated in 96% ethanol for PCR and sequencing, or in 4% PFA in PBS for histological examinations and fluorescence *in situ* hybridization (FISH). Additionally, adult specimens of *Dasytes virens* were collected in Aken, Germany, on May 20, 2011, as well as in the vicinity of Cursdorf, Germany, on June 13, 2015, and stored in 70% ethanol.

### DNA Extraction, PCR, Cloning, and Sequencing of Bacterial Symbiont 16S rRNA

For molecular characterization of the symbionts localized in the bacteriome of *D. plumbeus*, eight individuals were dissected in sterile water, after fixation and surface-sterilization in 70% ethanol. The bacteriomes were identified according to earlier descriptions and dissected with sterile forceps. DNA was extracted separately from the bacteriomes of each individual, respectively, using the MasterPure^TM^ DNA purification Kit (Epicentre Technologies) according to the manufacturer’s instructions, including a lysozyme treatment (1.3 mg/ml final concentration) for 30 min at 37°C, and stored in 20 μl 0.1 M Tris/HCl. Likewise, DNA was extracted from three complete individuals of *D. virens* collected in Aken, using the same protocol, as well as from the dissected bacteriomes of six male and six female *D. virens* collected in Cursdorf.

Almost complete 16S rRNA amplicons of the bacterial symbionts of *D. plumbeus* and *D. virens* were obtained by PCR with general eubacterial primers fD1 and rP2 (Weisburg et al., 1991) (**Table 1**). PCRs were performed on a Biometra T-Professional thermocycler in total reaction volumes of 25 μl containing 10 mM Tris-HCl, 50 mM KCl, 0.1% Triton X-100, 2.5 mM MgCl_2_, 240 μM deoxynucleoside triphosphates, 20 μmol of each primer, 1 U of Taq DNA polymerase (Roboklon, Berlin) and 2 μl of template. Cycle parameters were as follows: 3 min at 94°C, followed by 32 cycles of 94°C for 40 s, 65°C for 1 min, and 72°C for 1 min, and a final extension time of 4 min at 72°C. PCR products were purified with the innuPREP Gel Extraction Kit (Analytik Jena) and subsequently cloned into *E. coli* with the CloneJET PCR Cloning Kit (Thermo Scientific) according to the manufacturer’s instructions. Eight positive clones were picked for each species and directly added to the PCR master mix, and plasmid inserts were amplified using the flanking primers M13F and M13R with the same PCR conditions as described above, except that the annealing temperature was set to 55°C. Purified PCR products were sequenced with primers rP2, M13R, M13F, Com1, and Klebs.250f to obtain full-length amplicon sequences (**Table 1**). Sequences were curated manually and assembled in Geneious R6 (Biomatters Ltd.).

**Table 1:** Primers and FISH probes used for identification and localization of symbionts.

Primer name	Sequence (5′–3′)	Fwd/rev	5′ mod.	Target	Reference
fD1	AGAGTTTGATCCTGGCTCAG	Fwd		Eubacteria	Weisburg et al., 1991
rP2	ACGGCTACCTTGTTACGACTT	Rev		Eubacteria	Weisburg et al., 1991
M13F	CAGGAAACAGCTATGAC	Fwd		Eubacteria	Boutin-Ganache et al., 2001
M13R	GTAAAACGACGGCCAG	Rev		Eubacteria	Boutin-Ganache et al., 2001
Com1	CAGCAGCCGCGGTAATAC	Fwd		Eubacteria	Schwieger and Tebbe, 1998
Klebs.250f	CAGCCACACTGGAACTGAGA	Fwd		*Klebsiella* spp. 16S	Sudakaran et al., 2012
Dasy_Sym_fwd2	CCTGGTCTTGACATCCGTAG	Fwd		*Dasytes* symbiont	This study
Dasy_Sym_rev2	GCGACGTATTTTATGAGATCTGC	Rev		*Dasytes* symbiont	This study
Dasy_ent-Cy5	CCAATGGTTATCCCCCTCCA	Rev	Cy5	*Dasytes* symbiont	This study
EUB388-Cy3	GCTGCCTCCCGTAGGAGT	Rev	Cy3	Eubacteria	Amann et al., 1990

In order to assess infection prevalence of symbionts in *D. plumbeus* and *D. virens*, the specific primers Dasy_Sym_fwd2 and Dasy_Sym_rev2 (**Table 1**) were designed based on the obtained 16S rRNA sequence and used for diagnostic PCR. For *D. virens*, DNA extracted from entire adult beetles (11 males, 11 females, and three of unknown sex) was subjected to diagnostic PCR, while bacteriome DNA extracts (*n* = 5, sex unknown) were used for *D. plumbeus*. *E. coli* K12 was used as a negative control to ensure specificity of the PCR. PCR setup and conditions were the same as described above, except that the annealing temperature was set to 62°C.

### Phylogenetic Analysis

Curated symbiont 16S rRNA sequences were aligned using the SINA aligner (Pruesse et al., 2012), and phylogenetic relationships were reconstructed using approximately-maximum-likelihood algorithms as implemented in FastTree 2.1.3 (Price et al., 2010). The general time-reversible (GTR) model was used, and *Pseudomonas fluorescens* and *Pseudomonas aeruginosa* were defined as outgroup to root the tree. The Shimodaira-Hasegawa test was used to obtain local support values for the nodes, based on 1,000 resamples.

### Bacterial Community Profiling by High-Throughput Sequencing

In order to characterize the bacterial communities associated with *D. plumbeus* and *D. virens*, pooled DNA extracts were prepared from eight bacteriomes of *D. plumbeus* (unsexed, pooled to be sequenced as one sample), from three adult *D. virens* collected in Aken (unsexed, pooled for sequencing), as well as from two replicates of male and female *D. virens* bacteriomes (from Cursdorf), respectively, consisting of three bacteriomes each. DNA samples were sent to an external service provider for high-throughput bacterial tag-encoded FLX amplicon sequencing (MR DNA, Shallowater, TX, USA), using 16S rRNA primers Gray28F (5′-GAGTTTGATCNTGGCTCA-3′) and Gray519R (5′-GTNTTACNGCGGCKGCTG-3′) (Sun et al., 2011).

A sequencing library was generated through one-step PCR with 30 cycles, using the HotStarTaq Plus Master Mix Kit (Qiagen, Valencia, CA, USA) and the following conditions: 94°C for 3 min, followed by 28 cycles of 94°C for 30s; 53°C for 40 s and 72°C for 1 min; after which a final elongation step at 72°C for 5 min was performed. Following PCR, all amplicon products from different samples were mixed in equal concentrations and purified using Agencourt Ampure beads (Agencourt Bioscience Corporation, Beverly, MA, USA). Sequencing extended from Gray28F, using a Roche 454 FLX instrument with Titanium reagents. Quality control and analysis of 454 reads was done in QIIME 1.9.1 (Caporaso et al., 2010). Low-quality ends of the sequences were trimmed with a sliding window size of 50 and an average quality cut-off of 25. Subsequently, all low quality reads (quality cut-off = 25) and sequences <200 bp were removed. Potential chimeras were detected using usearch61 by *de novo* chimera detection (Edgar et al., 2011) and removed from further analysis. The remaining high-quality reads were then clustered into operational taxonomic units (OTUs) using a multiple OTU picking strategy with cdhit (Li and Godzik, 2006) and uclust (Edgar, 2010), with 97% similarity cut-offs, respectively. For each OTU, the longest sequence was chosen as representative sequence. RDP classifier (Wang et al., 2007) and BLASTn against the NCBI database were used for taxonomy assignment. A representative 16S rRNA sequence of the *Dasytes* symbiont obtained by Sanger sequencing was included in the database for taxonomy assignment, in order to assess abundance of symbiont reads in the 454 dataset. An OTU table was generated describing the occurrence of bacterial phylotypes within the samples. OTUs were combined on the genus level to summarize relative abundances.

### Symbiont Localization by Fluorescence *In situ* Hybridization (FISH)

Based on the obtained symbiont 16S rRNA sequences, the specific oligonucleotide probe Dasy_ent-Cy5 was designed for localization of the symbionts in *D. plumbeus* through FISH on tissue preparations (whole-mount) as well as in semithin sections. For whole-mount FISH, the digestive tract and reproductive organs of three PFA-fixated female beetles were dissected and washed three times in 0.3% Triton X-100 in PBS. Following permeabilization in 70% acetic acid for 1 min at 60°C, the samples were incubated in hybridization buffer (0.9 M NaCl, 0.02 M Tris/HCl pH 8.0, 0.01% SDS) for 30 min at 60°C. Hybridization was then achieved by incubation for 16 h at 60°C in 100 μl hybridization buffer containing 5 μl of the symbiont-specific probe Dasy_ent-Cy5 (500 nM) and the general eubacterial probe EUB338-Cy3 (500 nM), respectively, as well as 5 μg/ml DAPI for counterstaining of host cell nuclei. Afterward, the specimens were washed twice in wash buffer (0.1 M NaCl, 0.02 M Tris/HCl pH8.0, 0.01% SDS, 5 mM EDTA) for 2 h at 60°C each, and twice in dH_2_O for 30 min at 60°C each, and subsequently mounted on microscope slides and embedded in VectaShield (Vector, Burlingame, CA, USA). Images were acquired using an AxioImager.Z1 fluorescence microscope (Zeiss, Jena, Germany).

For higher resolution of symbiont-bearing structures, FISH was also performed on semithin sections of *D. plumbeus* as described previously (Kaltenpoth et al., 2012; Sudakaran et al., 2012). Briefly, a single adult individual was embedded in Technovit 8100 (Heraeus Kulzer, Wehrheim, Germany), and semithin sections (8 μm) were obtained on a microtome (Microm HM355S) with a glass blade and transferred to silanized microscope slides (Marienfeld). Samples were hybridized for 90 min at 60°C in the same hybridization mix as described for the whole-mount FISH. Two wash steps with pre-warmed washing buffer (composition see above), the second for 20 min at 60°C, as well as rinsing with dH_2_O served to remove residual probe. After drying at room temperature, slides were covered with VectaShield and inspected on an AxioImager.Z1 fluorescence microscope (Zeiss, Jena, Germany).

### 3D-Reconstruction of Symbiont-Bearing Organs

For three-dimensional reconstruction of the digestive tract and the associated symbiont-bearing organs, an adult female of *D. plumbeus* was embedded in epoxy resin (Epoxy embedding kit, Sigma). Semithin sections (2 μm) were obtained on a microtome (Microm HM355S) with a diamond blade and transferred to silanized microscope slides (Marienfeld). Samples were stained with a filtered toluidine blue/pyrimidine solution (0.4% toluidine blue, 0.1% pyrimidine G and 0.4% di-sodium-tetraborate in water) for 2 min at 60°C, washed briefly in water, air-dried, treated briefly with xylol, and then embedded in Entellan (Merck). Images of all sections were acquired on an AxioImager.Z1 and aligned with Fiji (Schindelin et al., 2012). For this, a TrakEM2 and an automatic alignment was generated. This data set was loaded into Amira 5.4.1 (Fei, Hillsboro, OR, USA) for 3D reconstruction.

### Data Accessibility

High-throughput bacterial 16S rRNA amplicon sequencing data for *D. plumbeus* and *D. virens* are available in the SRA of NCBI under accession number SRP083132 (BioProject ID PRJNA340363, comprising BioSamples SAMN05712926-31). Almost complete 16S rRNA sequences of ‘*Candidatus* Dasytiphilus stammeri’ from *D. plumbeus* and *D. virens* are available under NCBI accession numbers KX784547-KX784552.

## Results

### Bacterial Symbionts of *Dasytes plumbeus* and *D. virens*

**FIGURE 1:**
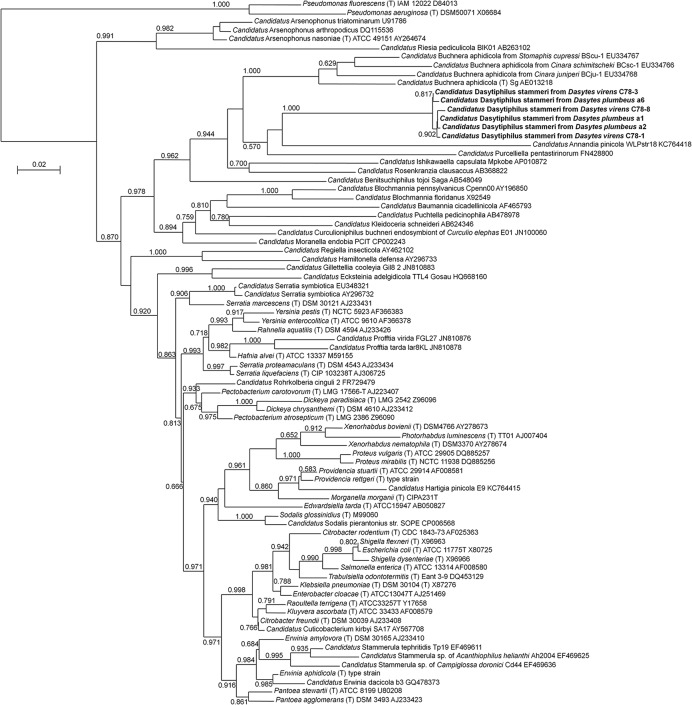
**Phylogenetic affiliation of ‘*Candidatus* Dasytiphilus stammeri’ within the Enterobacteriaceae.** Phylogenetic relationships were reconstructed with an approximately-maximum-likelihood algorithm as implemented in FastTree 2.1.3 (Price et al., 2010) using the GTR model. Local support values from 1,000 resamples are given at the nodes.

For the identification of bacterial symbionts associated with the two *Dasytes* species, DNA was extracted from entire beetles or from dissected bacteriomes and subjected to general eubacterial PCRs and subsequent cloning and sequencing. Samples of both species consistently yielded gamma-proteobacterial sequences that were related to other intracellular symbionts in insects. Phylogenetic analyses using almost complete 16S rRNA gene sequences placed the symbionts of *D. plumbeus* and *D. virens* in a monophyletic clade most closely related to ‘*Candidatus* Annandia pinicola,’ ‘*Candidatus* Purcelliella pentastirinorum,’ and ‘*Candidatus* Buchnera aphidicola,’ the intracellular symbionts of adelgids, fulgoroid planthoppers, and aphids, respectively (**Figure 1**). Interestingly, however, the *Dasytes* endosymbionts only showed 83.4–87.4% identity on the 16S rRNA level to *Annandia, Purcelliella*, and *Buchnera*, while sequence similarity within the *Dasytes* symbiont clade was between 99.1 and 99.7%. Furthermore, there were no consistent differences between the sequences of symbionts from *D. plumbeus* and *D. virens*, respectively. We propose the candidate species name ‘*Candidatus* Dasytiphilus stammeri’ for the endosymbionts of *Dasytes plumbeus* and *D. virens* (for a description of the new taxon, see below). Diagnostic PCR revealed the presence of ‘*Ca.* D. stammeri’ in 100% of the tested male (*n* = 11) and female (*n* = 11) *D. virens*, as well as in the bacteriomes of *D. plumbeus* (*n* = 5), indicating a consistent association of both species and both sexes with ‘*Ca*. D. stammeri.’

### Microbiota Associated with *Dasytes plumbeus* and *D. virens*

In order to gain a more comprehensive overview of the microbial communities associated with *D. plumbeus* and *D. virens*, DNA extracts from both beetle species were subjected to high-throughput sequencing of bacterial 16S rRNA amplicons. Even though the relative abundances of bacterial taxa detected by this approach have to be interpreted with caution due to possible PCR amplification biases, it allows for a broad survey of the host-associated bacterial diversity. For the two *Dasytes* species, FLX sequencing resulted in 7,600 to 46,503 bacterial 16S rRNA reads per sample after quality-filtering and chimera-checking (mean ± SD = 17,285 ± 14,624 per sample). The sequences were binned into 283 OTUs based on a 97% similarity cut-off. Most of the abundant OTUs were affiliated with the Enterobacteriaceae, with the majority of these being most closely related to ‘*Candidatus* Dasytiphilus stammeri.’ Collectively, *Dasytiphilus*-related reads amounted to 52.4–98.7% of the sequences in *D. plumbeus* and *D. virens*, irrespective of whether dissected bacteriomes or entire beetles were subjected to DNA extraction and microbiota profiling (**Table 2**). Apart from *Dasytiphilus*, no other taxon was consistently detected across individuals of the two species, but individual samples showed infection with *Rickettsia*, *Spiroplasma, Kocuria*, or an Enterobacteriaceae taxon closely related to *Erwinia* and *Citrobacter* (**Table 2**).

**Table 2:** Bacterial community associated with *Dasytes plumbeus* (*n* = 1) and *D. virens* (*n* = 5) as revealed by bacterial tag-encoded FLX sequencing of 16S rRNA amplicons.

Taxon	*Dasytes plumbeus*	*Dasytes virens*				
		Cursdorf1	Cursdorf2	Cursdorf3	Cursdorf4	Aken
Actinobacteria; *Kocuria*	4.5	0.0	0.0	0.0	0.0	0.0
Alphaproteobacteria; *Rickettsia*	0.0	0.0	0.0	45.6	0.0	0.0
**Gammaproteobacteria; *Dasytiphilus***	**72.7**	**96.1**	**93.4**	**52.4**	**98.7**	**96.6**
Gammaproteobacteria; *Erwinia/Citrobacter*	20.6	0.0	0.0	0.0	0.0	0.0
Mollicutes; *Spiroplasma*	0.0	0.0	0.7	0.1	0.4	3.4
Others	2.2	3.9	6.0	2.0	0.9	0.1
Total number of high-quality sequences	46,503	9,704	7,600	11,045	12,296	16,564

*Given are relative abundances of the most abundant genera, with the intracellular symbiont ‘Candidatus Dasytiphilus stammeri’ highlighted in bold font. Samples consisted of pooled bacteriomes for D. plumbeus and the D. virens samples from Cursdorf (Cursdorf1–2: males; Cursdorf3–4: females), and entire adult beetles for the *D. virens* sample from Aken.*

### Localization of Bacterial Symbionts in *Dasytes*

**FIGURE 2:**
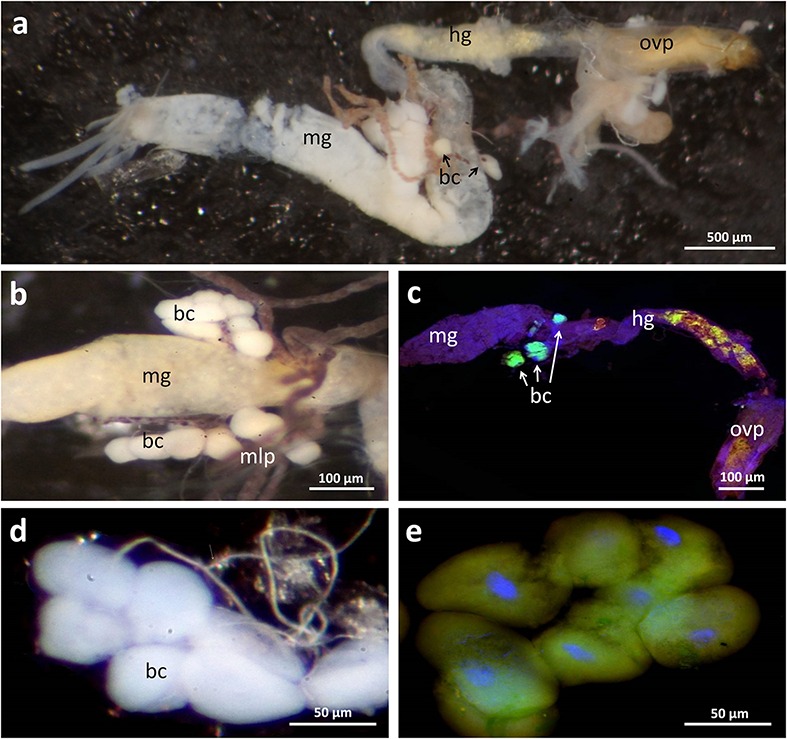
**Localization of bacteriomes associated with the digestive tract and the Malpighian tubules in *Dasytes virens.* (a)** Overview image of the dissected digestive tract. **(b)** Close-up of the bacteriomes located at the posterior end of the mid-gut. **(c)** Whole mount *in situ* hybridization micrograph on a dissected gut. Symbionts were specifically stained with Dasy_Ent-Cy5 (green), while the general eubacterial probe EUB388-Cy3 (red) and DAPI (blue) were used for counterstaining. Note the strong fluorescent signal in the bacteriomes, and the autofluorescence in the hind-gut originating from pollen grains. **(d,e)** Enlarged bright-field **(d)** and fluorescence **(e)** micrograph of an individual bacteriome. Scale bars represent 500 μm **(a)**, 100 μm **(b,c)**, and 50 μm **(d,e)**, respectively. Abbreviations: mg, mid-gut; mlp, Malpighian tubules; hg, hind-gut; bc, bacteriocytes; ovp, ovipositor (surrounding the gut).

**FIGURE 3:**
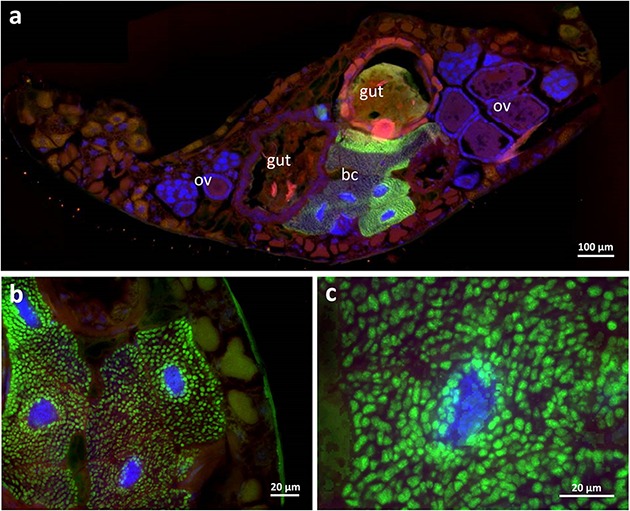
**Fluorescence *in situ* hybridization of ‘*Candidatus* Dasytiphilus stammeri’ in the bacteriomes of a female *Dasytes plumbeus.* (a)** Cross-section through the entire abdomen of *D. plumbeus*, and **(b,c)** close-ups of bacteriocytes. Symbionts were specifically stained with Dasy_Ent-Cy5 (green), while the general eubacterial probe EUB388-Cy3 (red) and DAPI (blue) were used for counterstaining. Abbreviations: bc, bacteriocytes; ov, ovaries.

During the dissection of adult beetles, the three mulberry-shaped cell clusters associated with the gut that were described by Holmgren (1902) and later by Stammer (1933) and Buchner (1965) could be easily identified. Each bacteriome is composed of five to ten polymorphic and strongly enlarged bacteriocytes (up to 80 μm in length). Using fluorescence *in situ* hybridization and histological sections, these cell clusters were found to harbor intracellular symbionts (**Figure 2**), confirming Stammer’s (1933) and Buchner’s (1965) earlier conclusion, which contradicted Holmgren’s (1902) interpretation that these cells constitute oenocytes. With a specific oligonucleotide probe designed based on the *Dasytiphilus* 16S sequence, the symbionts were identified as ‘*Ca*. D. stammeri’ (**Figure 3**). The symbionts densely populated all three bacteriomes as globular or slightly elongate cells of approximately 2–4 μm in size (**Figure 3c**).

**FIGURE 4:**
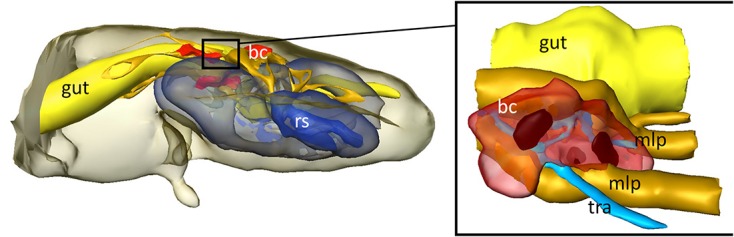
**3D-reconstruction of the interior organs in the abdomen of *D. plumbeus*.** mlp, Malpighian tubules; bc, bacteriocytes; tra, tracheae; rs, reproductive system.

**FIGURE 5:**
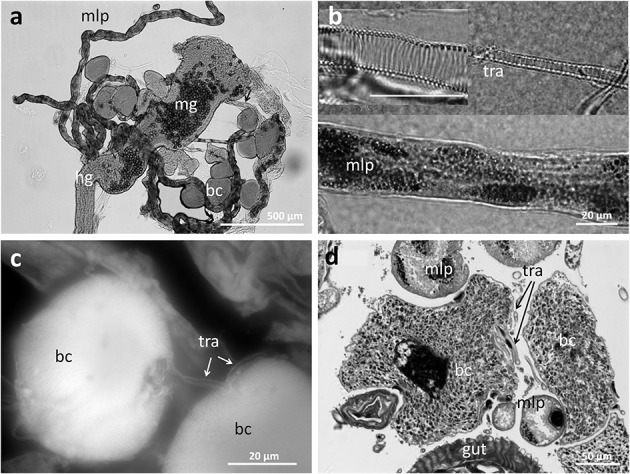
**Structure of the bacteriomes in *Dasytes* and their connection to the respiratory system. (a)** Bacteriomes localized at the junction of mid- and hind-gut. Note the pollen grains in the mid-gut. **(b)** Close-up images of trachea and Malpigian tubule closely associated with the bacteriome. **(c)** Close association of two bacteriocytes with a trachea. Image created based on Cy3 autofluorescence. **(d)** Cross section of a bacteriome in *Dasytes plumbeus*, with the densely packed intracellular symbionts clearly visible. Note the close association with Malpighian tubules and tracheae. Abbreviations: mg, mid-gut; mlp, Malpighian tubules; hg, hind-gut; bc, bacteriocytes; tra, tracheae.

In order to obtain more detailed information on the localization and organization of the bacteriomes, we prepared semithin (2 μm) section series for light microscopy and three-dimensional reconstruction. Both 3D-reconstructions and light microscopic investigations confirmed the close association of the bacteriomes to the gut as well as the Malpighian tubules (**Figures 4** and **5**). Furthermore, the bacteriomes are well provided with trachea (**Figures 4** and **5**), as is observed for the bacteriomes of diverse insects (Buchner, 1965).

## Discussion

Bacterial mutualists are widespread in insects and can convey a range of novel ecological traits to their hosts (Feldhaar, 2011). Here, we report on the association of beetles in the genus *Dasytes* with the intracellular mutualist ‘*Ca*. Dasytiphilus stammeri’ that is closely related to the gamma-proteobacterial primary endosymbionts of aphids, adelgids, and fulgoroid planthoppers.

The family Dasytidae comprises around 1,500 species of herbivorous beetles, many of which have a carnivorous larval stage (Mirutenko, 2013). Unfortunately, nothing is known about bacterial symbionts in the entire superfamily Cleroidea, which comprises, e.g., the families Malachiidae, Melyridae, and Cleridae, in addition to the Dasytidae (Hunt et al., 2007; Bocakova et al., 2012). However, the close phylogenetic affiliation of *‘Ca.* D. stammeri’ with the intracellular symbionts of plant sap sucking Hemiptera suggests horizontal exchange of symbionts or acquisition of closely related ancestors. It should be noted, however, that conclusions based on phylogenetic analyses of 16S rRNA sequences have to be considered with caution, due to the limited resolution as well as possible long-branch attraction problems that can lead to erroneous groupings of derived taxa (Bergsten, 2005).

In addition to ‘*Ca*. D. stammeri,’ several other bacterial taxa were detected in *Dasytes* samples by high-throughput bacterial 16S rRNA amplicon sequencing, including *Rickettsia*, *Spiroplasma, Kocuria*, and an Enterobacteriaceae taxon closely related to *Erwinia* and *Citrobacter. Rickettsia* and *Spiroplasma* are widespread insect symbionts that can have mutualistic effects on their hosts (e.g., Lukasik et al., 2013), but more commonly cause pathogenicity or reproductive manipulation (Weinert et al., 2007; Russell et al., 2012). The Enterobacteriaceae taxon is likewise closely related to pathogenic bacteria including *Serratia* and *Klebsiella* and may thus represent an infection in the single individual containing this bacterium. Alternatively, contamination from the gut during dissection is conceivable, as Enterobacteriaceae often represent one of the dominant bacterial families in insects’ guts (Colman et al., 2012). However, the lack of Enterobacteriaceae other than ‘*Ca*. D. stammeri’ in the *D. virens* sample from Aken comprising of entire beetles (**Table 2**) indicates that this family is not generally abundant in the gut of *Dasytes*. Finally, little is known about the impact of *Kocuria* on insect hosts, even though members of this genus have been repeatedly detected in insect guts, e.g., of the bark beetle *Dendroctonus rhizophagus* (Morales-Jimenez et al., 2012), the emerald ash borer *Agrilus planipennis* (Vasanthakumar et al., 2008), and the tobacco hornworm *Manduca sexta* (van der Hoeven et al., 2008). None of these bacterial taxa was consistently detected in individuals of *D. plumbeus* and *D. virens*, so they have to be considered facultative associates of these beetles.

In addition to the consistent presence of ‘*Ca*. D. stammeri’ across *Dasytes* individuals, its intracellular localization within bacteriomes that are connected to the posterior part of the mid-gut supports its mutualistic nature. In other insect taxa, symbionts localized in the gut or gut-associated caeca are known to provision limiting B-vitamins (Salem et al., 2014) or amino acids to the host (Feldhaar et al., 2007; Vigneron et al., 2014), contribute to the break-down of fastidious dietary polymers (Engel et al., 2012; Brune, 2014), provide colonization resistance against pathogens or parasites (Koch and Schmid-Hempel, 2011), or detoxify plant secondary metabolites (Dowd, 1989; Dowd and Shen, 1990) or even synthetic pesticides used in human agriculture (Kikuchi et al., 2012). However, functions requiring enzymatic activity in the gut are so far limited to extracellularly localized symbionts, possibly due to the difficulty of transporting bacterial enzymes across host membranes. In *Dasytes*, the symbionts may contribute essential amino acids or B vitamins to the host, which can be limiting in pollen (Roulston and Cane, 2000). Alternatively, given the close connection of the bacteriomes with the gut through a short duct, an enzymatic contribution toward the break down or penetration of the pollen exine and/or intine is conceivable. However, most individuals still contained at least partial pollen grains in the hind-gut (**Figure 2c**), which may indicate penetration of the pollen walls to acquire the nutritious cytoplasmic content, rather than complete digestion of the fastidious polymers (Roulston and Cane, 2000). Symbiont-assisted break down of pollen walls has been recently suggested for honeybees, based on the metagenomic discovery of bacterial pectate lyases (Engel et al., 2012).

The dietary change from larval carnivory to adult pollen-feeding makes beetles of the genus Dasytes an interesting taxon to investigate life-stage specific symbiont contributions to host metabolism. Future functional investigations based on genomic analysis or experimental perturbation of the *Dasytes* symbiosis may reveal the symbiont-provided benefits to the host and yield interesting new insights into the mechanistic basis of pollen digestion in insects.

### Description of ‘*Candidatus* Dasytiphilus stammeri’

‘*Candidatus* Dasytiphilus stammeri’ [Da.sy.ti.phi’lus stam’me.ri; N.L. n. *Dasytes* (Coleoptera, Dasytidae) the generic name of the host organism; Gr. *philos* friend; N.L. masc. n. *Dasytiphilus*, mutualistic symbiont (friend) of beetles in the genus *Dasytes*. *Stammeri*, refers to the original description of the symbionts in *Dasytes* beetles by Hans Jürgen Stammer (Stammer, 1933)]. Uncultured, Gram-negative, non-motile, coccoid or short rod-shaped bacteria of approximately 2–4 μm in diameter that can be assigned to the Enterobacteriaceae (Gamma-Proteobacteria) on the basis of their 16S rRNA gene sequence. The bacteria live symbiotically within specialized bacteriomes associated with the digestive tract of adult beetles in the genus *Dasytes*. The 16S rRNA gene sequence of the symbionts of *D. plumbeus* and *D. virens* can be amplified diagnostically with primers Dasy_Sym_fwd2 (5′-CCTGGTCTTGACATCCGTAG-3′) and Dasy_Sym_rev2 (5′-GCGACGTATTTTATGAGATCTGC-3′). The symbionts are selectively stained with the FISH probe Dasy_ent-Cy5 (5′-Cy5-CCAATGGTTATCCCCCTCCA-3′).

## Author Contributions

BW and MK conceived of the study, and both authors collected specimens. BW conducted the molecular work and the 3D reconstruction, MK performed the QIIME analysis and reconstructed the symbiont phylogeny. Both authors wrote the manuscript.

## Conflict of Interest Statement

The authors declare that the research was conducted in the absence of any commercial or financial relationships that could be construed as a potential conflict of interest.

## References

[B1] Amann R. I. Binder B. J. Olson R. J. Chisholm S. W. Devereux R. Stahl D. A. (1990). Combination of 16S ribosomal RNA targeted oligonucleotide probes with flow-cytometry for analyzing mixed microbial populations. *Appl. Environ. Microbiol.* 56 1919–1925. 2200342 10.1128/aem.56.6.1919-1925.1990 PMC184531

[B2] Bergsten J. (2005). A review of long-branch attraction. *Cladistics* 221 163–193. 10.1111/j.1096-0031.2005.00059.x 34892859

[B3] Bocakova M. Constantin R. Bocak L. (2012). Molecular phylogenetics of the melyrid lineage (Coleoptera: Cleroidea). *Cladistics* 28 117–129. 10.1111/j.1096-0031.2011.00368.x 34861761

[B4] Boutin-Ganache I. Raposo M. Raymond M. Deschepper C. F. (2001). M13-tailed primers improve the readability and usability of microsatellite analyses performed with two different allele-sizing methods. *Biotechniques* 31 24–28. 11464515

[B5] Brune A. (2014). Symbiotic digestion of lignocellulose in termite guts. *Nat. Rev. Microbiol.* 12 168–180. 10.1038/nrmicro3182 24487819

[B6] Buchner P. (1965). *Endosymbiosis of Animals with Plant Microorganisms.* New York, NY: Interscience Publishers.

[B7] Caporaso J. G. Kuczynski J. Stombaugh J. Bittinger K. Bushman F. D. Costello E. K. (2010). QIIME allows analysis of high-throughput community sequencing data. *Nat. Methods* 7 335–336. 10.1038/nmeth.f.303 20383131 PMC3156573

[B8] Colman D. R. Toolson E. C. Takacs-Vesbach C. D. (2012). Do diet and taxonomy influence insect gut bacterial communities? *Mol. Ecol.* 21 5124–5137. 10.1111/j.1365-294X.2012.05752.x 22978555

[B9] Douglas A. E. (1989). Mycetocyte symbiosis in insects. *Biol. Rev. Camb. Philos. Soc.* 64 409–434. 10.1111/j.1469-185X.1989.tb00682.x 2696562

[B10] Douglas A. E. (2009). The microbial dimension in insect nutritional ecology. *Funct. Ecol.* 23 38–47. 10.1111/j.1365-2435.2008.01442.x

[B11] Dowd P. F. (1989). In situ production of hydrolytic detoxifying enzymes by symbiotic yeasts in the cigarette beetle (Coleoptera: Anobiidae). *J. Econ. Entomol.* 82 396–400. 10.1093/jee/82.2.396

[B12] Dowd P. F. Shen S. K. (1990). The contribution of symbiotic yeast to toxin resistance of the cigarette beetle (*Lasioderma serricorne*). *Entomol. Exp. Appl.* 56 241–248. 10.1111/j.1570-7458.1990.tb01402.x

[B13] Edgar R. C. (2010). Search and clustering orders of magnitude faster than BLAST. *Bioinformatics* 26 2460–2461. 10.1093/bioinformatics/btq461 20709691

[B14] Edgar R. C. Haas B. J. Clemente J. C. Quince C. Knight R. (2011). UCHIME improves sensitivity and speed of chimera detection. *Bioinformatics* 27 2194–2200. 10.1093/bioinformatics/btr381 21700674 PMC3150044

[B15] Engel P. Martinson V. G. Moran N. A. (2012). Functional diversity within the simple gut microbiota of the honey bee. *Proc. Natl. Acad. Sci. U.S.A.* 109 11002–11007. 10.1073/pnas.1202970109 22711827 PMC3390884

[B16] Feldhaar H. (2011). Bacterial symbionts as mediators of ecologically important traits of insect hosts. *Ecol. Entomol.* 36 533–543. 10.1111/j.1365-2311.2011.01318.x

[B17] Feldhaar H. Straka J. Krischke M. Berthold K. Stoll S. Mueller M. J. (2007). Nutritional upgrading for omnivorous carpenter ants by the endosymbiont *Blochmannia*. *BMC Biol.* 5:48 10.1186/1741-7007-5-48 PMC2206011 17971224

[B18] Flórez L. Biedermann P. H. W. Engl T. Kaltenpoth M. (2015). Defensive symbioses of animals with prokaryotic and eukaryotic microorganisms. *Nat. Prod. Rep.* 32 904–936. 10.1039/c5np00010f 25891201

[B19] Holmgren N. (1902). Über die Exkretionsorgane des Apion flavipes und *Dasytes niger*. *Anat. Anz.* 22 225–239.

[B20] Hosokawa T. Kikuchi Y. Shimada M. Fukatsu T. (2007). Obligate symbiont involved in pest status of host insect. *Proc. R. Soc. B Biol. Sci.* 274 1979–1984. 10.1098/rspb.2007.0620 PMC2275188 17567556

[B21] Hunt T. Bergsten J. Levkanicova Z. Papadopoulou A. John O. S. Wild R. (2007). A comprehensive phylogeny of beetles reveals the evolutionary origins of a superradiation. *Science* 318 1913–1916. 10.1126/science.1146954 18096805

[B22] Joy J. B. (2013). Symbiosis catalyses niche expansion and diversification. *Proc. R. Soc. B Biol. Sci.* 280 20122820 10.1098/rspb.2012.2820 PMC3574373 23390106

[B23] Kaltenpoth M. Yildirim E. Gürbüz M. F. Herzner G. Strohm E. (2012). Refining the roots of the beewolf-*Streptomyces* symbiosis: antennal symbionts in the rare genus *Philanthinus* (Hymenoptera, Crabronidae). *Appl. Environ. Microbiol.* 78 822–827. 10.1128/AEM.06809-11 22113914 PMC3264120

[B24] Kikuchi Y. Hayatsu M. Hosokawa T. Nagayama A. Tago K. Fukatsu T. (2012). Symbiont-mediated insecticide resistance. *Proc. Natl. Acad. Sci. U.S.A.* 109 8618–8622. 10.1073/pnas.1200231109 22529384 PMC3365206

[B25] Koch H. Schmid-Hempel P. (2011). Socially transmitted gut microbiota protect bumble bees against an intestinal parasite. *Proc. Natl. Acad. Sci. U.S.A.* 108 19288–19292. 10.1073/pnas.1110474108 22084077 PMC3228419

[B26] Li W. Godzik A. (2006). Cd-hit: a fast program for clustering and comparing large sets of protein or nucleotide sequences. *Bioinformatics* 22 1658–1659. 10.1093/bioinformatics/btl158 16731699

[B27] Lukasik P. van Asch M. Guo H. Ferrari J. Charles J. Godfray H. (2013). Unrelated facultative endosymbionts protect aphids against a fungal pathogen. *Ecol. Lett.* 16 214–218. 10.1111/ele.12031 23137173

[B28] McFall-Ngai M. Hadfield M. G. Bosch T. C. G. Carey H. V. Domazet-Loso T. Douglas A. E. (2013). Animals in a bacterial world, a new imperative for the life sciences. *Proc. Natl. Acad. Sci. U.S.A.* 110 3229–3236. 10.1073/pnas.1218525110 23391737 PMC3587249

[B29] Mirutenko V. V. (2013). The families Malachiidae and Dasytidae in the collections of the Goulandris Natural History Museum, Athens, Greece. *Entomol. Hell.* 22 1–6.

[B30] Morales-Jimenez J. Zuniga G. Ramirez-Saad H. C. Hernandez-Rodriguez C. (2012). Gut-associated bacteria throughout the life cycle of the bark beetle *Dendroctonus* rhizophagus Thomas and Bright (Curculionidae: Scolytinae) and their cellulolytic activities. *Microb. Ecol.* 64 268–278. 10.1007/s00248-011-9999-0 22234511

[B31] Moran N. A. (2007). Symbiosis as an adaptive process and source of phenotypic complexity. *Proc. Natl. Acad. Sci. U.S.A.* 104 8627–8633. 10.1073/pnas.0611659104 17494762 PMC1876439

[B32] Nakabachi A. Ueoka R. Oshima K. Teta R. Mangoni A. Gurgui M. (2013). Defensive bacteriome symbiont with a drastically reduced genome. *Curr. Biol.* 23 1478–1484. 10.1016/j.cub.2013.06.027 23850282

[B33] O’Connor R. M. Fung J. M. Sharp K. H. Benner J. S. McClung C. Cushing S. (2014). Gill bacteria enable a novel digestive strategy in a wood-feeding mollusk. *Proc. Natl. Acad. Sci. U.S.A.* 111 E5096–E5104. 10.1073/pnas.1413110111 25385629 PMC4250168

[B34] Price M. N. Dehal P. S. Arkin A. P. (2010). FastTree 2 – Approximately maximum-likelihood trees for large alignments. *PLoS ONE* 5:e9490 10.1371/journal.pone.0009490 PMC2835736 20224823

[B35] Pruesse E. Peplies J. Gloeckner F. O. (2012). SINA: accurate high-throughput multiple sequence alignment of ribosomal RNA genes. *Bioinformatics* 28 1823–1829. 10.1093/bioinformatics/bts252 22556368 PMC3389763

[B36] Roulston T. H. Cane J. H. (2000). Pollen nutritional content and digestibility for animals. *Plant Syst. Evol.* 222 187–209. 10.1007/BF00984102

[B37] Russell J. A. Funaro C. F. Giraldo Y. M. Goldman-Huertas B. Suh D. Kronauer D. J. C. (2012). A veritable menagerie of heritable bacteria from ants, butterflies, and beyond: broad molecular surveys and a systematic review. *PLoS ONE* 7:e51027 10.1371/journal.pone.0051027 PMC3527441 23284655

[B38] Salem H. Bauer E. Strauss A. S. Vogel H. Marz M. Kaltenpoth M. (2014). Vitamin supplementation by gut symbionts ensures metabolic homeostasis in an insect host. *Proc. R. Soc. B Biol. Sci.* 281:20141838 10.1098/rspb.2014.1838 PMC4213650 25339726

[B39] Schindelin J. Arganda-Carreras I. Frise E. Kaynig V. Longair M. Pietzsch T. (2012). Fiji: an open-source platform for biological-image analysis. *Nat. Methods* 9 676–682. 10.1038/nmeth.2019 22743772 PMC3855844

[B40] Schwieger F. Tebbe C. C. (1998). A new approach to utilize PCR-single-strand-conformation polymorphism for 16s rRNA gene-based microbial community analysis. *Appl. Environ. Microbiol.* 64 4870–4876. 9835576 10.1128/aem.64.12.4870-4876.1998 PMC90936

[B41] Shelomi M. Danchin E. G. J. Heckel D. G. Wipfler B. Bradler S. Zhou X. (2016). Horizontal gene transfer of pectinases from bacteria preceded the diversification of stick and leaf insects. *Sci. Rep.* 6:26388 10.1038/srep26388 PMC4876471 27210832

[B42] Simon C. Frati F. Beckenbach A. Crespi B. Liu H. Flook P. (1994). Evolution, weighting, and phylogenetic utility of mitochondrial gene sequences and a compilation of conserved polymerase chain reaction primers. *Ann. Entomol. Soc. Am.* 87 651–701. 10.1093/aesa/87.6.651

[B43] Stammer H. J. (1933). Neue Symbiosen bei Coleopteren. *Verh. Dtsch. Zool. Ges.* 35 150–155.

[B44] Sudakaran S. Retz F. Kikuchi Y. Kost C. Kaltenpoth M. (2015). Evolutionary transition in symbiotic syndromes enabled diversification of phytophagous insects on an imbalanced diet. *ISME J.* 9 2587–2604. 10.1038/ismej.2015.75 26023876 PMC4817627

[B45] Sudakaran S. Salem H. Kost C. Kaltenpoth M. (2012). Geographic and ecological stability of the symbiotic mid-gut microbiota in European firebugs, *Pyrrhocoris apterus* (Hemiptera, Pyrrhocoridae). *Mol. Ecol.* 21 6134–6151. 10.1111/mec.12027 23017151

[B46] Sun Y. Wolcott R. D. Dowd S. E. (2011). Tag-encoded FLX amplicon pyrosequencing for the elucidation of microbial and functional gene diversity in any environment. *Methods Mol. Biol.* 733 129–141. 10.1007/978-1-61779-089-8_9 21431767

[B47] Tsuchida T. Koga R. Fukatsu T. (2004). Host plant specialization governed by facultative symbiont. *Science* 303 1989–1989. 10.1126/science.1094611 15044797

[B48] van der Hoeven R. Betrabet G. Forst S. (2008). Characterization of the gut bacterial community in *Manduca sexta* and effect of antibiotics on bacterial diversity and nematode reproduction. *FEMS Microbiol. Lett.* 286 249–256. 10.1111/j.1574-6968.2008.01277.x 18647359

[B49] Vasanthakumar A. Handelsman J. Schloss P. D. Bauer L. S. Raffa K. F. (2008). Gut microbiota of an invasice subcortical beetle, *Agrilus planipennis* Fairmarine, across various life stages. *Environ. Entomol.* 37 1344–1353. 10.1093/ee/37.5.1344 19036215

[B50] Vigneron A. Masson F. Vallier A. Balmand S. Rey M. Vincent-Monegat C. (2014). Insects recycle endosymbionts when the benefit is over. *Curr. Biol.* 24 2267–2273. 10.1016/j.cub.2014.07.065 25242028

[B51] Wang Q. Garrity G. M. Tiedje J. M. Cole J. R. (2007). Naive Bayesian classifier for rapid assignment of rRNA sequences into the new bacterial taxonomy. *Appl. Environ. Microbiol.* 73 5261–5267. 10.1128/AEM.00062-07 17586664 PMC1950982

[B52] Weinert L. A. Tinsley M. C. Temperley M. Jiggins F. M. (2007). Are we underestimating the diversity and incidence of insect bacterial symbionts? A case study in ladybird beetles. *Biol. Lett.* 3 678–681. 10.1098/rsbl.2007.0373 17878145 PMC2111056

[B53] Weisburg W. G. Barns S. M. Pelletier D. A. Lane D. J. (1991). 16s ribosomal DNA amplification for phylogenetic study. *J. Bacteriol.* 173 697–703. 1987160 10.1128/jb.173.2.697-703.1991 PMC207061

